# *Myrmecia*, Not *Asterochloris*, Is the Main Photobiont of *Cladonia subturgida* (*Cladoniaceae*, *Lecanoromycetes*)

**DOI:** 10.3390/jof9121160

**Published:** 2023-12-02

**Authors:** Raquel Pino-Bodas, Miguel Blázquez, Asunción de los Ríos, Sergio Pérez-Ortega

**Affiliations:** 1 Biodiversity and Conservation Area, Department of Biology and Geology, Physics and Inorganic Chemistry, Rey Juan Carlos University, C/Tulipán s/n, 28933 Móstoles, Spain; 2 Royal Botanic Gardens, Kew, Richmond, London TW9 3DS, UK; 3 Department of Mycology, Real Jardín Botánico (CSIC), 28014 Madrid, Spain; m.blazquezva@rjb.csic.es (M.B.); sperezortega@rjb.csic.es (S.P.-O.); 4 Museo de Ciencias Naturales (CSIC), 28006 Madrid, Spain; arios@mncn.csic.es

**Keywords:** *Cladonia*, lichenized fungi, phycobiont, symbiosis, Trebouxiophyceae, ultrastructure

## Abstract

This study explores the diversity of photobionts associated with the Mediterranean lichen-forming fungus *Cladonia subturgida*. For this purpose, we sequenced the whole ITS rDNA region by Sanger using a metabarcoding method for ITS2. A total of 41 specimens from Greece, Italy, France, Portugal, and Spain were studied. Additionally, two specimens from Spain were used to generate four cultures. Our molecular studies showed that the genus *Myrmecia* is the main photobiont of *C. subturgida* throughout its geographic distribution. This result contrasts with previous studies, which indicated that the main photobiont for most *Cladonia* species is *Asterochloris*. The identity of *Myrmecia* was also confirmed by ultrastructural studies of photobionts within the lichen thalli and cultures. Photobiont cells showed a parietal chloroplast lacking a pyrenoid, which characterizes the species in this genus. Phylogenetic analyses indicate hidden diversity within this genus. The results of amplicon sequencing showed the presence of multiple ASVs in 58.3% of the specimens studied.

## 1. Introduction

Lichen-forming fungi are adapted to obtain their carbon source from the algae and/or the cyanobacteria with which they associate, thus becoming obligate symbionts [1]. About 17% of all fungi and 27% of known Ascomycota are lichen-forming fungi [2], indicating the evolutionary success of this life strategy, which appears in most terrestrial ecosystems. About 90% of the green algae present in lichen symbioses belong to the class Trebouxiophyceae in Chlorophyta. Among the most frequent genera are *Asterochloris*, *Coccomyxa*, *Myrmecia*, and *Trebouxia* [3,4]. In addition, species of *Trentepohliales* in the class Ulvophyceae also frequently act as photobionts, usually associated with distant lineages of lichen-forming fungi such as *Arthoniomycetes*, *Lichinomycetes*, *Dothidiomycetes*, *Ostropales* in *Lecanoromycetes*, and *Pyrenulales* in *Eurotiomycetes* [4]. Despite recent advances based on DNA sequencing studies, the diversity of algae associated with lichen-forming fungi, the photobionts, is still far from being well known. The identity of photobionts has only been studied in approximately 5% of lichens up to now [1,4,5]. Significant progress has been made in recent years with the description of several new genera and species of photobionts [6,7,8,9,10,11], suggesting that the true diversity of photobionts may be much greater than previously thought [4,5].

Photobionts play an important role in the adaptation of lichens to the environment, and the selection of a photobiont affects their fitness [12]. Rambold [13] and Yahr et al. [14] have studied mycobiont-photobiont interactions in terms of specificity and selectivity. Thus, specificity refers to the range of compatible photobiont lineages, while selectivity refers to the frequency of association among the compatible lineages. Regarding specificity, there are various levels of specificity among lichen-forming fungi. On the one hand, species showing high specificity would be associated with a small number of algal lineages [15,16]. On the other hand, species that associate with a wide range of photobiont lineages are considered to have low specificity [17,18,19]. However, interactions between mycobionts and photobionts can involve great complexity, and in some cases, the degree of specificity may vary across the distributional range of a mycobiont [20]. Lichen-forming fungi commonly use a strategy of locally preferentially selecting certain photobionts among compatible ones that are believed to be better adapted to local environmental conditions. [14,21,22]. Thus, a mycobiont species can be associated with different photobionts under different conditions of temperature, humidity, elevation, or soil type [12,23,24]. Although some studies exploring patterns of photobiont diversity throughout the distribution range of a lichen-forming fungal species have been carried out [17,21,25,26] and others analyzing photobiont shifts under an environmental gradient or in populations living in different habitats [13,23,27,28,29,30,31], we still know little about how photobiont diversity is structured across the host distribution and how the turnover of photobionts allows the mycobiont to tolerate a wide range of environmental conditions [23]. Thus, understanding the diversity and association patterns between mycobionts and photobionts is pivotal for unraveling the amplitude of the ecological niche and the evolutionary dynamics of lichens [32].

*Cladonia* is among the genera of macrolichen-forming fungi with the highest number of species [2]. It is primarily terricolous, with a sub-cosmopolitan distribution [33]. Numerous studies have been dedicated to exploring the diversity of photobionts associated with *Cladonia* [24,34,35,36,37,38,39,40,41]. Most of these studies find that *Cladonia* species associate with species of the green algal genus *Asterochloris*, although photobionts of other genera of *Trebouxyophyceae* have been found to be sporadically associated with *Cladonia* species [42,43,44]. Different levels of specificity and selectivity have been identified among *Cladonia* species [13,35,41,45], with mycobiont identity, reproductive mode, climate, geography, and soil features being the factors that best explain the patterns of genetic diversity of photobionts [24,39,41].

The current research centers on examining photobiont diversity within *Cladonia subturgida* Samp., a prevalent species in the Mediterranean region that thrives across a range of environments, including open *Quercus* forests and *Cistus* shrubs with acidic soil, spanning from the Thermo- to Supra-Mediterranean belts [46]. Species distribution models suggest that *Cladonia subturgida* could have a wider distribution, including the Canary Islands [47]. *Cladonia subturgida* is characterized by a persistent and dominant primary thallus, which often lacks a secondary thallus, with a prevalence of asexual reproduction. The upper surface of its squamules is greenish to olive green, and the underside is whitish purple towards the edges. The infrequent podetia are branched near the apex and present open axils [46,48].

This study aims to characterize the photobionts associated with *C. subturgida* by molecular and ultrastructural analyses. Based on previous studies of photobionts associated with *Cladonia*, we expected that *C. subturgida* was associated with different species of *Asterochloris*. Surprisingly, we found *Myrmecia* in a population of *C. subturgida* from Spain, which led us to explore the diversity of photobionts associated with this species throughout its distribution.

## 2. Materials and Methods

### 2.1. Sampling, Isolation, and Culture of Photobionts

A selection of specimens (n = 39) from Pino-Bodas et al. [47,48] was made to study the photobionts associated with *C. subturgida* (Table 1). We selected specimens from a wide range of geographical regions to represent as complete a distribution of the species as possible. Additionally, new specimens of *C. subturgida* (n = 2) were collected to isolate the photobionts and conduct ultrastructural studies (Table 1). For isolating and culturing the photobionts, a single squamule per specimen was selected. The photobionts were isolated following Muggia et al. [49] with small modifications. In summary, the squamules were washed three times each, for 15 min, using miliQ water, then the fragments were washed using 1:10 of Tween20 solution for 30 min, and finally washed twice using miliQ water for 15 min. Clean lichen fragments were homogenized with a pestle in 2 mL of miliQ water, and 50 μL of suspension was spread in the Petri dish using an inoculation loop. The photobionts were cultured in solid Bold’s Basal Medium [50], and the Petri dishes were sealed with Parafilm. The cultures were incubated at 20 °C with a light–dark regime (14:10 h) and a light intensity of 60–100 μmol photons m^–2^s^–1^. Algal colonies of 2–3 mm in size were subcultured in new Petri dishes with the same medium for long-term growth. The identity of the cultured photobionts was confirmed by picking the colonies for DNA extraction and sequencing of the ribosomal cistron ITS rDNA.

### 2.2. DNA Extraction, Amplification, and Sequencing

The genomic DNA was extracted using the E.Z.N.A. forensic kit (OMEGA Biotek, Norcross, Georgia, USA) following the manufacturer’s instructions. The DNA was eluted in 100 μL of the elution buffer (Tris-Cl 10 mM) included in the kit. The ITS rDNA region was amplified with SSU-1780A/ITS4 [34] with the PCR conditions described in Pino-Bodas and Stenroos [41]. The new sequences were deposited in GenBank (OR760202-OR760210).

In addition, we used a metabarcoding approach to explore the photobiont diversity within *Cladonia subturgida* thalli. Following previous studies [23], the ITS2 region was used as a barcode. The primer pairs FDGITS2-f and FDGITS2-r [23], with Fluidigm CS1 and CS2 universal oligomer sequences at their 5′ ends, were used for amplification. PCR reactions were carried out in a total volume of 15 μL, containing 3 μL of template DNA, 0.3 μL of each primer (10 μM), 7 μL of ACCUZYME™ Mix (2x), which contains the high-fidelity ACCUZYME™ DNA polymerase (Bioline, Sydney, Australia), and 4.4 μL of distilled water. The PCR settings consisted of an initial denaturation at 95 °C for 1 min, 35 cycles of 95 °C for 15 s, 54 °C for 15 s, and 72 °C for 15 s, with a final extension at 72 °C for 5 min. PCR products were checked in 1% agarose gels stained with SYBR™ Safe DNA Gel Stain (Thermo Fisher Scientific, Alcobendas, Spain), quantified using the Qubit dsDNA HS (High Sensitivity) Assay Kit (Thermo Fisher Scientific, Alcobendas, Spain), and pooled in equimolar concentrations for sequencing on c. 1/25 of a MiSeq run (Illumina, San Diego, CA, USA). Sequencing was carried out by the RTSF Genomics Core at Michigan State University (East Lansing, MI, USA).

### 2.3. Sequence Processing

Raw sequence data were processed using the DADA2 pipeline [51] in R 4.2.2 [52], using the parameters described in [53]. In short, DADA2 takes a set of demultiplexed paired-end fastq files, filters the sequences based on their quality and length, assembles them into error-corrected amplicon sequence variants (ASVs), and removes chimeric ASVs. Following Dal Grande et al. [23], we excluded ASVs with less than 100 reads from downstream analyses since they were most likely exogenous to the symbiosis. We then assigned taxonomy to the ASVs via BLAST searches [54]. All sequences obtained in this study are available in the SRA (NCBI) under BioProject PRJNA1033642.

### 2.4. Phylogenetic Analyses

To explore the phylogenetic placement of the *Myrmecia* ASVs within the genus, we generated phylogenetic hypotheses based on both maximum likelihood and Bayesian inference. We constructed a dataset comprising the sequences of the five most prevalent ASVs, the nine sequences derived from cultures (three) and thalli (six), and thirty-five ITS rDNA sequences that are representative of the genus diversity accessible in GenBank. The selection of GenBank sequences was based on the BLAST searches. Most of them were generated by [44,55,56,57]. Three ITS sequences of *Lobosphaera incisa* [58], *Trebouxia lynnae* [59], and *Vulcanochloris guanchorum* [10] were used as outgroups. The GenBank accession numbers of all sequences are listed in Supplementary Table S1. The sequences were aligned using the program MAFFT v7.450 [60] as implemented in Geneious Prime^®^ v2023.2. We set the following parameters: the FFT-NS-I x1000 algorithm, a gap open penalty of 1.53, the 200PAM/k = 2 scoring matrix, and an offset value of 0.123. We used RAxML [61] to find the best-scoring maximum likelihood tree and performed 1000 rapid bootstrap pseudoreplicates to evaluate nodal support. MrBayes 3.2.7 [62,63] was used to infer phylogenetic relationships using a Bayesian framework. The analysis started with a random tree, and two simultaneous, parallel four-chain runs were executed over 1 × 10^7^ generations and sampled after every 1000th step. The first 20% of data was removed as burn-in. The 50% majority-rule consensus tree was calculated from the remaining trees. Nodes with bootstrap values equal to or higher than 70% and with posterior probabilities equal to or higher than 95% were considered to be significantly supported. Both maximum likelihood and Bayesian analyses were run in the CIPRES Science Gateway [64].

### 2.5. Transmission Electron Microscopy

The ultrastructural study was carried out on the squamules of four specimens and cultures of photobionts isolated from *C. subturgida*. The specimens studied belonged to *C. humilis* s.l. (according to Pino-Bodas et al. [65]), *C. foliacea*, and *C. subturgida* (Table S2), all of them growing together in the same locality. The samples for Transmission Electron Microscopy (TEM) were prepared according to the protocol described in de los Ríos and Ascaso [66]. Briefly, small fragments of lichen thalli or small clumps of cultured algal cells were first fixed in glutaraldehyde (3% *v*/*v* in phosphate buffer), then postfixed in osmium tetroxide (1% *w*/*v* in phosphate buffer), and finally dehydrated in a graded ethanol series before embedding in Spurr’s resin. Ultrathin sections were cut using a diamond knife on an Ultracut-E ultramicrotome (Reichert, Wetzlar, Germany) and subsequently stained with uranyl acetate and lead citrate. Images were captured using a JEM-2100 transmission electron microscope (JEOL, Tokyo, Japan) at the CNB-CSIC facility.

### 2.6. Haplotype Networks and Statistical Analyses

For the *Myrmecia* ASVs, a haplotype network was constructed in PopART 1.7 [67] under statistical parsimony, selecting the TCS method [68].

Non-metric multidimensional scaling (NMDS) ordination using Bray–Curtis dissimilarities was used to compare intrathalline photobiont composition across the distribution of *C. subturgida*. To test significant differences among countries (France, Greece, Italy, and Spain) and regions in the Mediterranean Basin (west, central, and east Mediterranean), permutation analysis of variance (PERMANOVA) was carried out with the *adonis2* function with 999 permutations. The analyses were implemented in the *vegan* R package [69]. To identify the ASVs driving geographical structure, species indicator analyses [70] were conducted. The *multpatt* function of the *indicspecies* R package [71] was used to assess significant associations of ASVs with different countries or regions. The analyses were carried out with 999 permutations. A Mantel test was carried out in *vegan* to detect a putative correlation between the genetic diversity of the main photobionts and the geographical distance. The geographical distance matrix was calculated using Euclidean distances between sites. To construct the main photobiont matrix, the ASV with the highest number of reads in each specimen was selected. The Mantel test was calculated using the Pearson correlation coefficient, and significance was computed using 2000 random permutations. Then, a partial Mantel test was used to assess correlations between the ITS rDNA genetic distance matrix of mycobionts and the main photobiont distance matrix, corrected with geographical distances. 

## 3. Results

### 3.1. Molecular Diversity

The BLAST searches revealed that all ITS rDNA sequences obtained by Sanger sequencing, both from thalli and from cultures, belonged to the genus *Myrmecia* (MH70374, *Myrmecia* sp. identity = 98.5%; OL625167, *Myrmecia* sp. identity = 99%).

**Figure 1:**
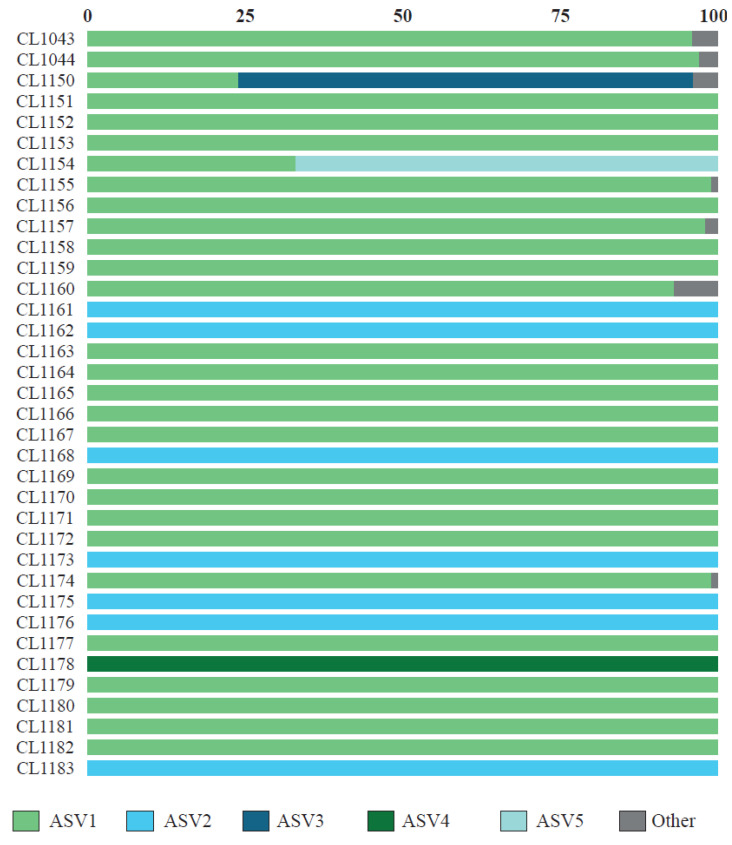
Relative abundance of the photobiont ASV recovered in individual thalli of *C. subturgida*.

From the metabarcoding study, we obtained a total of 482,279 raw reads of ITS2, of which 313,282 (65%) passed the DADA2 quality filter, obtaining an average of 8702 ± 4755 reads per specimen. After removing ASVs represented by less than 100 reads, 19 ASVs remained. The results of the BLAST searches are summarized in Table 2. The first five ASVs, which accounted for 98% of the reads and acted as the main photobiont in all studied specimens (Figure 1), belonged to the genus *Myrmecia*. The remaining ASVs belonged to *Trebouxia* spp. (nine ASVs), *Asterochloris mediterranea* (one ASV), *Vulcanochloris symbiotica* (one ASV), *Coccomyxa* sp. (one ASV), *Hemichloris* (one ASV), and one unidentified alga with values of percent identity below 90% with the closest named taxa (two ASVs). The richness of ASVs per thallus ranged from one to seven; more than one photobiont ASV was found in 58.3% of thalli (Figure 1). In 34 specimens, a single ASV was represented by >96% of all reads, and it belonged to *Myrmecia*. In only two specimens (CL1150 and CL1154), we observed the co-occurrence of two *Myrmecia* ASVs. In these specimens, the most abundant ASV was represented by 66% and 72% of reads, respectively, while the second more abundant photobiont was represented by 34% and 28% of reads, respectively. In seven specimens, we also observed the presence of non-*Myrmecia* photobionts (Figure 1). However, a predominant ASV belonging to *Myrmecia* was observed in all cases.

### 3.2. Phylogenetic Relationships

**Figure 2:**
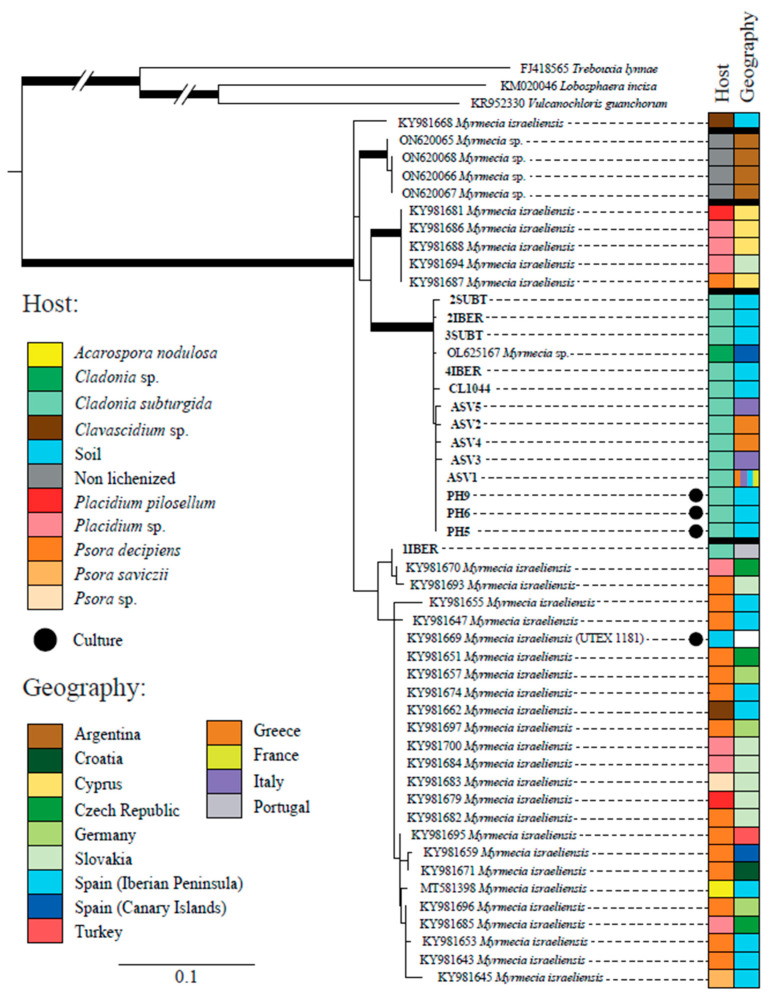
Phylogenetic tree based on the ITS region and inferred by maximum likelihood (RAxML) showing the relationships among different lineages within the genus *Myrmecia*. Branches with bootstrap values ≥ 70% and posterior probability ≥95% are highlighted in bold. The table on the right side shows the host and geographic origin of all accession numbers. The meaning of the color schemes in the table is explained at the left of the figure.

All the sequences obtained during this study but 1IBER (from a thallus collected in Portugal) formed a well-supported clade together with one sequence obtained from the GenBank (OL625167) from *Cladonia* sp. (Figure 2). Sequences of *Myrmecia* available on GenBank have been obtained from terrestrial lichens, mainly from Europe but also from Argentina and Turkey. Most of them have been attributed to *Myrmecia israeliensis*, forming at least four distinct clades, three of which were strongly supported.

### 3.3. Photobiont Ultrastructure

**Figure 3:**
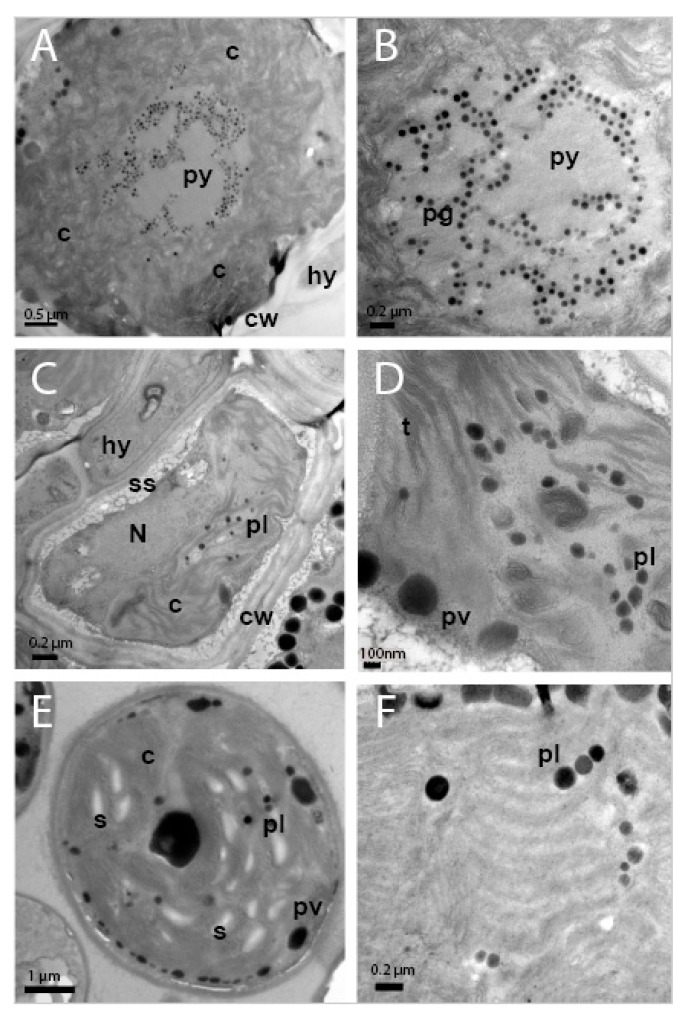
TEM images showing the ultrastructure of photobiont cells in the thalli of *Cladonia* ssp. and culture. (**A**) Photobiont cell in *Cladonia humilis* s.lat. thallus showing a central chloroplast (c), with pyrenoid (py) and cell wall (cw), in the proximity of a fungal hyphae (hy). (**B**) Detail of pyrenoid (py) showing pyrenoglobuli (pg). (**C**) Photobiont cell in the thallus of *Cladonia subturgida*, surrounded by hyphae (hy), showing a parietal chloroplast (c) without pyrenoid, the nucleus (N), plastoglobuli (pl), cell wall (cw) and secretory space (ss). (**D**) Detail of the chloroplast showing the thylakoids (t), plastoglobuli (pl) and peripheral vesicles (pv). (**E**) Photobiont cell from *Cladonia subturgida* in culture showing a parietal chloroplast (c) without pyrenoid, plastoglobuli (pl), peripheral vesicules (pv), and starch granules (s). (**F**) Detail of the chloroplast of a cultured photobiont cell showing the presence of plastoglobuli (pl) between the thylakoids.

TEM analysis showed that the cellular ultrastructure of photobiont cells from *C. subturgida* thalli, as well as that of cultured photobionts, was different from the one observed in photobiont cells of *C. foliacea* (not shown) and *C. humilis* thalli. In the algal layer of *C. foliacea* and *C. humilis* thalli (Figure 3A,B), photobiont cells showed a central chloroplast with a clear pyrenoid (py) harboring pyrenoglobuli (pg). Pyrenoglobuli were observed in association with curved thylakoid tubules located inside the pyrenoid. These pyrenoid features resembled those of the *irregularis* type of Friedl [72], typical of *Asterochloris* species [6,8,9]. However, photobiont cells in *Cladonia subturgida* thalli showed a parietal chloroplast without a pyrenoid, which occupied most of the cell (Figure 3C). Electron-dense globules (plastoglobuli) and starch granules were observed between the chloroplast thylakoids (Figure 3D). Similar chloroplast ultrastructural features were found in culture photobiont cells (Figure 3E,F). Starch granules were more frequent in cultured photobiont cells than in photobiont cells inside the lichen thallus (Figure 3C–E).

### 3.4. Genetic Structure of the Photobiont Associated with C. subturgida

**Figure 4:**
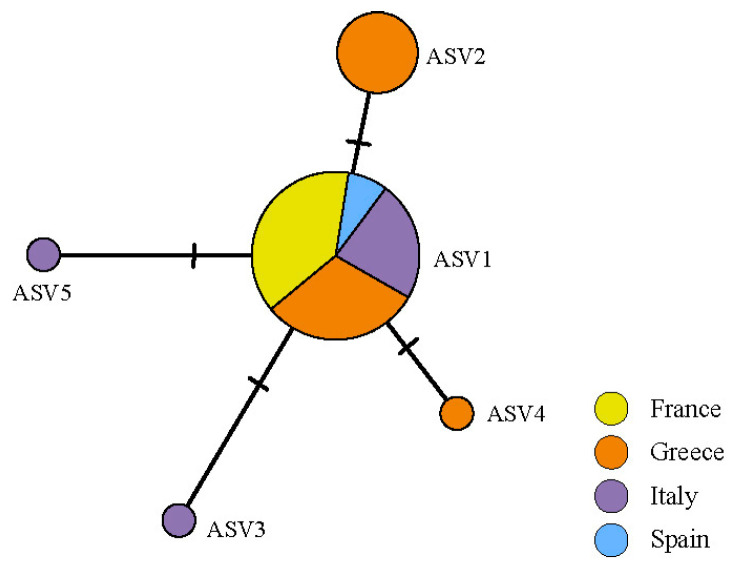
Haplotype network of *Myrmecia* ASVs found in the thalli of *C. subturgida*, inferred in PopArt under the TCS algorithm. Each circle represents an ASV and the circle size is proportional to the ASV frequency.

The ASVs belonging to *Myrmecia* formed a single haplotype network (Figure 4). ASV1 was the most frequent, present in all countries sampled. The second most common was ASV2, separated by two mutations from ASV1 and restricted to Greece. The other three ASVs belonging to *Myrmecia* were restricted, two to Italy and one to Greece (Figure S1).

**Figure 5:**
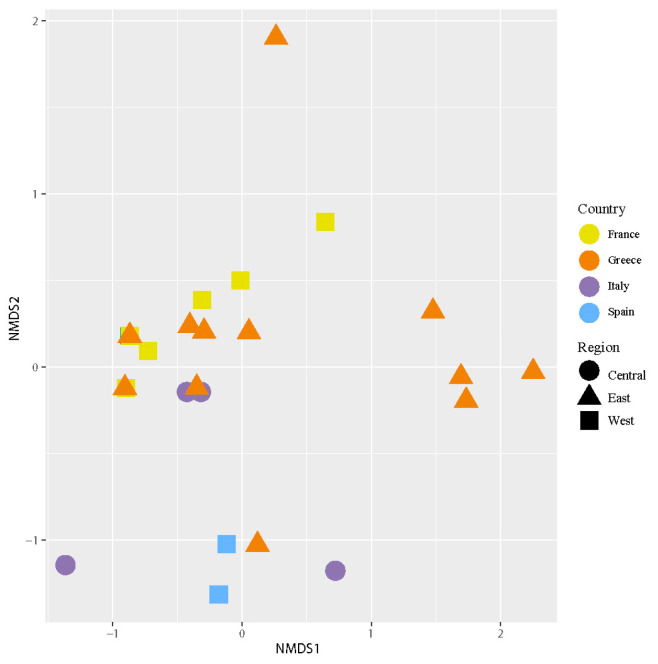
Nonmetric multidimensional scaling (NMDS) of intrathalline photobiont composition in *C. subturgida*. Different symbols represent specimens collected in different regions (west, central, and east Mediterranean), and different colors represent samples collected in different countries (France, Italy, Greece, and Spain). The stress of the ordination was 0.08.

The NMDS (Figure 5) analysis revealed different groups of samples examined according to their intrathalline photobiont diversity. PERMANOVA analyses found significant differences in intrathalline photobiont composition among specimens from different countries (F = 3.2152, R2 = 0.23161, *p* = 0.02) and different regions (F = 4.0856, R2 = 0.19847, *p* = 0.019). Three main clusters were observed, two of them having a broad distribution across the Mediterranean region, while the third cluster comprises specimens restricted to Greece.

The species indicator analyses identified a total of six significant ASVs (Table 3). The ASV2 showed a significant association with Greece, whereas the others were associated with Spain. When the ASVs were analyzed by region, the species indicator test identified a single significant ASV (ASV2, statistics = 0.521, *p*-value = 0.016), which was associated with the east Mediterranean region.

The Mantel test detected a correlation between the main photobiont matrix distance and geographical distances (r = 0.07048, *p*-value = 0.036482), but no correlation was detected between the mycobiont and photobiont distance matrices (r = −0.2121, *p*-value = 0.97251).

## 4. Discussion

This study demonstrates that *C. subturgida* is associated with *Myrmecia* sp. as the main photobiont throughout its distribution range, confirming our preliminary results based on a population from Spain. This result is supported by molecular and ultrastructural data. The genus *Myrmecia*, encompassing nine species [73], comprises both lichenized and free-living species [74]. Species of this genus are characterized by their coccoid cells with parietal chloroplasts, which do not contain a pyrenoid [75]. These characteristics are shared by the *Myrmecia* species found in *C. subturgida* (Figure 3). *Myrmecia* is a polyphyletic genus [74,76], and our phylogenetic results based on ITS rDNA indicate that the *Myrmecia* strains associated with *C. subturgida* are related to *M. israeliensis* (Figure 2), a lineage related to *Asterochloris* [76,77]. Our results revealed a probably hidden diversity within *M. israeliensis*, representing a species complex with at least four distinct clades. However, further studies, morphological, physiological, and phylogenetic, will be necessary to clarify the taxonomy of the genus.

The association of *Cladonia* with *Myrmecia* is not unexpected, as other genera of lichen-forming fungi that associate with *Asterochloris*, such as *Heteroplacicium, Placidium*, and *Psora*, can also lichenize with *Myrmecia* [28,55,78,79]. In addition, Vančurová et al. [10] found a specimen of *Cladonia* sp. associated with *Myrmecia* in the Canary Islands. In general, *Myrmecia* has been found associated with terricolous lichen species, a habitat also shared by *C. subturgida*. The presence of free-living *Myrmecia* in the soils of regions with extreme climates, such as the Namib Desert [80] or high mountain ecosystems [57,81], has led to the hypothesis that this genus is well adapted to extreme arid conditions [57]. Although the conditions under which *C. subturgida* thrives are not so extreme, its climatic optimum is in areas with low rainfall and prolonged summer drought [47]. This suggests that the association with *Myrmecia* could be an adaptive advantage for *C. subturgida*.

The association of *Cladonia* species with photobionts other than *Asterochloris* had been previously reported [43,82,83]. In some cases, photobiont shifts are associated with highly disturbed areas [43]. This flexibility would allow the mycobiont to colonize habitats under highly stressful conditions where the availability of the preferential photobiont might be low or it could be subject to limited ecological performance. However, we do not consider this to be the case for the *Myrmecia-C. subturgida* association found in this study, since *Myrmecia* represented the main photobiont in all the analyzed samples, indicating that it is the preferred photobiont of this species. The specimens analyzed were not restricted to a locality or small geographic area whose environmental conditions may differ from the average niche of the species, but on the contrary, they were quite distributed within the distribution of *C. subturgida*, covering the common habitats where *C. subturgida* lives [46].

Re-synthesis experiments demonstrated the ability of *Cladonia cristatella* to associate with *Myrmecia israelensis*, with clear connections between the fungus and the algae, even forming pre-squamules [83]. This indicates that *Myrmecia* is a compatible photobiont with some species of *Cladonia*. These findings seem to indicate that mycobiont-photobiont recognition signals might be quite conserved in evolution, so that they are at least partially unrestrictive among closely related algae [84,85]. The presence of several phylogenetically related intrathalline photobionts also seems to support this hypothesis [30]. Our knowledge of the recognition signals between mycobionts and photobiota is still scarce. It appears that the secretion of fungal lectins and arginases is key to the recognition of compatible photobionts, while the secretion of cyclic peptides has been observed in several photobionts [86]. In re-synthesis experiments between *Cladonia grayi* and *Asterochloris glomerata*, an increased expression of membrane transporter proteins has been observed. In addition, the photobiont expresses genes encoding for extracellular hydrolases, and the mycobiont expresses ammonium and ribitol transporters [87].

Our results contrast with previous studies carried out on *Cladonia.* The photobionts of a large number of *Cladonia* species have been examined from a wide range of geographical and climatic regions [34,39,40,41,44], and *Asterochloris* was found to be the only associated photobiont genus. Moya et al. [55] highlighted that the diversity and presence of *Myrmecia* as a photobiont may have been underestimated due to the general primers used in photobiont studies, which may not effectively target this genus, even when it acts as the main photobiont. However, we do not consider this to be the case for *Cladonia* because: (1) *Myrmecia* has not been found in previous metabarcoding studies from other *Cladonia* species [88,89,90] and (2) the commonly used primers employed in this study allowed amplification of *Myrmecia* by Sanger sequencing in all the thalli of *C. subturgida* studied. This observation implies that the primers used in this study, as in prior *Cladonia* studies [34,41], effectively target the *Myrmecia* species.

*Cladonia subturgida*, phylogenetically closely related to *C. rangiformis*, is a member of the Rangiformes subclade within the larger clade *Cladonia* [91]. The photobionts associated with most of the species in this subclade have been studied and identified as members of the genus *Asterochloris* [6,34,41]. Thus, the association between *C. subturgida* and *Myrmecia* seems to be unique within the *Rangiformes* subclade. It is noteworthy that none of the species commonly co-occurring with *C. subturgida*, such as *C. cervicornis*, *C. firma*, *C. foliacea*, *C. humilis*, and *C. rangiformis* [47], use *Myrmecia* as a photobiont. This observation effectively rules out the possibility of *Myrmecia* serving as a highly specialized photobiont for this *Cladonia* community. The ultrastructural analyses carried out in this study show that species coexisting with *C. subturgida* are associated with *Asterochloris* as the main photobiont (Figure 3). Therefore, within the genus *Cladonia*, *C. subturgida* appears to exhibit a marked specialization by forming a distinctive and highly selective association with *Myrmecia*. *Cladonia subturgida* displays a pronounced preference for ASV1, which is present throughout its distribution (Figure 1 and Figure 4).

### 4.1. Photobiont Diversity within C. subturgida Thalli

The first molecular studies on photobionts began to show that more than one photobiont genotype could co-occur within the lichen thallus [92,93,94,95]. Currently, metabarcoding studies are revealing that this is a common phenomenon, as it has been found in thalli of phylogenetically disparate lichen-forming fungi [23,53,96,97,98,99,100]. In 58.3% of the analyzed thalli, more than one photobiont was found (Figure 1). However, in most of them, a single *Myrmecia* ASV represents over 96% of the reads. In only two of the analyzed specimens did we find a secondary photobiont (as defined by Paul et al. [97] and Dal Grande et al. [23]), with 23% and 32.9% of all reads, respectively. In both cases, the secondary ASV also belongs to *Myrmecia*. This shows that, similarly to what happens in other species [23], a low percentage of *C. subturgida* specimens have multiple photobiont genotypes in high proportion. However, in other species, such as *Ramalina farinacea*, it seems to be a dominant phenomenon [99], with most specimens showing two dominant photobionts with different physiological behavior [101,102], which could imply an adaptive advantage in changing environmental conditions [21].

Metabarcoding studies can also give us an idea of the range of compatible photobionts in the mycobiont. Thus, our results could indicate that *C. subturgida* might be able to establish symbiosis with four different genera: *Asterochloris*, aff. *Hemichloris, Myrmecia, Trebouxia*, and *Vulcanochloris*. Nevertheless, except for *Myrmecia*, the presence of the other genera is quite marginal. To establish with confidence that these algae are indeed forming associations with the mycobiont and are not the result of contamination from cortical biofilms, it is imperative to employ alternative verification methods [21]. *Asterochloris mediterranea* is the most common photobiont species in association with *Cladonia* in both the Mediterranean region and Macaronesia [44,56], detected in seven specimens of *C. subturgida*, which leads us to think that it might not be a contamination. *Trebouxia* ASVs were detected in 18 specimens, a result that agrees with those found in metabarcoding studies in other *Cladonia* species [88,90,103]. This, together with recent findings indicating that under certain conditions some *Cladonia* species have a preference to associate with *Trebouxia* [43], could support the hypothesis of the acquisition of diverse photobionts during thallus development [104,105], so that, if environmental conditions change, the mycobiont could survive by allowing the better-adapted photobiont to increase its proportion rapidly [98].

The presence of *Coccomyxa* and aff. *Hemichloris* as *Cladonia* photobionts is uncertain. *Hemichloris antarctica* is considered part of the cryptoendolithic microbial community [106,107], and although it has been found in lichen thalli of various species in low proportion [98,108,109], most authors do not consider this species to be a possible photobiont. Similarly, the ASV of *Coccomyxa* accounts for less than 1% of the total reads. *Coccomyxa* acts as a photobiont in other genera of lichen-forming fungi [4], while only metabarcoding studies [88,103] have found it associated with *Cladonia*. However, re-synthesis experiments suggest that *Cladonia* does not show parasitic behavior towards *Coccomyxa* as it does towards other incompatible algae [84].

*Vulcanochloris* has only been found to date in association with *Stereocaulon* [10,40,110]. However, this genus probably is a photobiont compatible with *Cladonia*, given its close phylogenetic relationships with *Asterochloris* and *Myrmecia* [10] and between *Stereocaulon* and *Cladonia* [111].

To summarize, it appears that there exists a pool of phylogenetically related algae that can act as temporary photobionts during early development [104,105]. These semi-compatible photobionts would not be completely replaced by the preferred photobiont but would remain in the thallus at low levels and may play a biological role in other developmental stages. In the case of corals, it has been demonstrated that these pseudocompatible symbionts appear to be key to holobiont stability, providing environmental resilience [112]. The putative biological role of these semi-compatible photobionts in lichen thalli is still unknown and should be tested in future studies.

### 4.2. Pattern of Genetic Diversity of Photobionts Associated with C. subturgida

Our phylogenetic analyses indicate that all *Myrmecia* ASVs form a single lineage; only one has been found associated with *Cladonia* to date. Only one Sanger sequence (1IBER) obtained from a thallus appears in another phylogenetically closely related clade (Figure 2). It reflects a low phylogenetic diversity of the main photobionts associated with *C. subturgida*. This pattern of high specificity is not very frequent and contrasts with most studies, where one lichen-forming fungi species is associated with multiple lineages of photobionts [17,22,25,28], particularly in a broad geographical context such as the Mediterranean region. However, caution should be exercised since, as indicated by Moya et al. [55], the *Myrmecia* photobionts are still poorly studied, and this lineage could establish symbiosis with other lichen-forming fungi.

Some of the known cases of reciprocal specificity or high specificity have been associated with the asexual mode of reproduction [113], including the genus *Cladonia* [35,114,115]. This explanation could be valid to explain the high specificity of *C. subturgida*, as its predominant reproduction is asexual by dispersal of thallus fragments [47]. The high specificity of species with asexual reproduction is assumed to be due to the dispersion of symbionts. However, our results show that the genetic structure of the symbionts is not congruent. While *Myrmecia* appears to be geographically structured, the genetic structure of *C. subturgida* estimated by Pino-Bodas et al. [47] did not show a geographic pattern. Therefore, the high reciprocal specificity could reflect either ancient co-dispersal events or that co-dispersal exists, but once fragments have become established, photobiont shifts occur [116].

Although ASV1 is the main photobiont in 72.2% of the thalli analyzed and is widely distributed throughout the Mediterranean region (Figure 4), the Mantel test indicated a geographic structure of the main photobionts. When the diversity of intrathalline photobionts was analyzed, a similar result was found. PERMANOVA results suggested a non-random distribution of the ASVs in the lichen thalli, showing a geographical distribution. According to the species indicator analyses, this geographical structure is largely determined by ASV2, which is restricted to Greece and was the main photobiont in 26.12% of the specimens studied. Other ASVs were also significantly associated with specimens from Spain. This also supports the hypothesis of local photobiont acquisitions during thallus development [104,105]. The geographic structure of *C. subturgida* photobionts is broadly congruent with that found in other Mediterranean lichens [99,109,117].

## 5. Conclusions

Throughout its geographical distribution, *Cladonia subturgida* is associated with *Myrmecia*, a photobiont rarely associated with *Cladonia*. The intrathalline photobiont diversity found was low, with most specimens containing only one ASV belonging to *Myrmecia*, which represented the majority of reads. The distribution of *Myrmecia* ASVs shows a geographical structure, although a dominant ASV is widespread throughout the Mediterranean region.

## Figures and Tables

**Table 1:** Specimens of *C. subturgida* used in this study with voucher information.

Code	Country	Locality	Collector and Collection	Long	Lat	Altitude	Vegetation and Substrate
CL1170	France	Provence-Alpes-Côte D’Azur, Var, Esterel Massif, Fréjus-Saint-Raphaël, Le Trayas, Col des Lentisques	A. R. Burgaz s.n. (MACB 111396)	43°28′13″ N	006°54′30″ E	260 m	*Quercus suber*, *Pinus pinaster*, *Arbutus unedo*, *Pistacia lentiscus*, *Erica* sp., *Viburnum* sp.; acid substrate
CL1172	France	Provence-Alpes-Côte D’Azur, Var, Fréjus-Saint-Raphäel, Esterel Massif, Route du Gargalon	A. R. Burgaz s.n. (MACB 111368)	43°28′19″ N	006°45′27″ E	33 m	*Quercus suber*, *Pinus pinaster* and shrubs
CL1174	France	Provence-Alpes-Côte D’Azur, Var, Gonfaron, Massif des Mures, Forêt communale de Gonfaron	A. R. Burgaz s.n. (MACB 111387)	43°19′08″ N	006°19′19″ E	153 m	*Quercus suber*; acid substrate
CL1177	France	Provence-Alpes-Côte D’Azur, Var, Le Cannet-des-Maures, Plaine Maures, Bayonne	A. R. Burgaz s.n. (MACB 111390)	43°20′54″ N	006°25′40″ E	82 m	*Pinus pinea*, *Quercus suber*, and *Juniperus oxycedrus*; sandstone substrate
CL1167	France	Provence-Alpes-Côte D’Azur, Var, Le Plan-de-la-Tour, Massif des Maures, Les Pierrons	A. R. Burgaz s.n. (MACB 111395)	43°22′15″ N	006°32′52″ E	201 m	*Quercus suber* and shrubs; siliceous substrate
CL1166	France	Provence-Alpes-Côte D’Azur, Var, Sainte-Maxime, Massif del Maures, close to Col de Gratteloup	A. R. Burgaz s.n. (MACB 111391)	43°22′08″ N	006°35′59″ E	105 m	*Quercus suber* and shrubs; siliceous substrate
CL1163	France	Provence-Alpes-Côte D’Azur, Var, Sainte-Maxime, Massif del Maures, to Saint Martin	A. R. Burgaz s.n. (MACB 111393)	43°24′37″ N	006°34′35″ E	105 m	*Quercus suber* and shrubs; granitic substrate
CL1164	France	Provence-Alpes-Côte D’Azur, Var, Vidauban, La Bastide Rouge, Plaine del Maures	A. R. Burgaz s.n. (MACB 111394)	43°23′47″ N	006°27′38″ E	62 m	*Pinus pinea*, *Quercus suber*, and *Juniperus oxycedrus*; sandstone substrate
CL1169	France	Provence-Alpes-Côte D’Azur, Var, Vidauban, Massif des Maures, Langoustaou	A. R. Burgaz s.n. (MACB 111370)	43°22′32″ N	006°31′09″ E	145 m	*Quercus suber*; acid substrate
CL1160	France	Provence-Alpes-Côte D’Azur, Var, Collobrières, Massif des Maures, Les Hautes Vaudreches	A. R. Burgaz s.n. (MACB 111389)	43°15′39″ N	006°18′00″ E	308 m	*Quercus suber*; acid substrate
CL1181	Greece	Crete, Chania, Kantamos-Selino Chondros	A.R. Burgaz s.n. (MACB 111383)	35°20′13″ N	023°41′03″ E	520 m	Spiny shrubland, *Erica manipuliflora*, and *Arbutus unedo*; quartzitic substrate
CL1180	Greece	Crete, Heraklion, Malevizi, Marathos	A.R. Burgaz s.n. (MACB 111384)	35°20′54″ N	024°59′34″ E	492 m	*Arbutus unedo* and *Erica manipuliflora*; quartzitic substrate
CL1175	Greece	Macedonia-Thrace, Chalkidiki, Ágios Óros Peninsula, Nea Roda	A.R. Burgaz s.n. (MACB 111388)	40°22′23″ N	023°55′46″ E	40 m	Maquis shrubland “frigana” and *Quercus coccifera*; gneiss and sandstone substrate
CL1168	Greece	Macedonia-Thrace, Chalkidiki, Polygyros, 17A road to Peleokastro	A.R. Burgaz s.n. (MACB 111380)	40°24′28″ N	023°25′13″ E	620 m	*Quercus coccifera*, deciduous *Quercus*, *Juniperus oxycedrus*, and *Erica arborea*; gneiss substrate
CL1178	Greece	Macedonia-Thrace, Chalkidiki, Polygyros, Settlement Agias, Anastasias road 16, 32 km	A.R. Burgaz s.n. (MACB 111373)	40°29′09″ N	023°11′51″ E	240 m	*Quercus coccifera*; sandstone substrate
CL1171	Greece	Macedonia-Thrace, Chalkidiki, Sithonia Peninsula, Agios Nikolaus, Karidi a Sarti	A.R. Burgaz s.n. (MACB 111371)	40°00′16″ N	023°53′10″ E	100 m	*Quercus coccifera*, *Pinus halepensis* and *Erica arborea*; granite substrate
CL1173	Greece	Macedonia-Thrace, Chalkidiki, Sithonia Peninsula, Torini, 73.8 km to Pórto Cárras	A.R. Burgaz s.n. (MACB 111385)	40°00′16″ N	023°53′10″ E	25 m	Shrubs with *Quercus coccifera*, *Pistacia lentiscus* and *Olea europea;*
CL1162	Greece	Macedonia-Thrace, Dio-Olympos, Leptokarya, Elassona-Leptokarya road	A.R. Burgaz s.n. (MACB 111377)	40°02′07″ N	022°30′59″ E	390 m	*Quercus rotundifolia*, *Juniperus oxycedrus* and *Erica* sp.; quartzite substrate
CL1176	Greece	Macedonia-Thrace, Evros, Dadia, entrada a la reserva forestal Dadia	A.R. Burgaz s.n. (MACB 111369)	41°07′25″ N	026°13′35″ E	90 m	*Pinus pinea*; quartzite substrate
CL1179	Greece	Macedonia-Traia, Island of Thasos, Theologos	A.R. Burgaz s.n. (MACB 111374)	40°39′16″ N	024°41′02″ E	220 m	*Quercus coccifera* and *Cistus*; substrate with schists and limestone
CL1158	Greece	Peloponese, Arcadia, Gortynia, Langadia, Kaloneri, Olympia-Tripoli roads	A.R. Burgaz s.n. (MACB 111379)	37°39′16″ N	022°04′42″ E	1003 m	*Abies* forest and *Juniperus oxycedrus*; basic substrate
CL1157	Greece	Peloponese, Arcadia, Gortynia, Langadia, Kaloneri, Olympia-Tripoli roads	A.R. Burgaz s.n. (MACB 111376)	37°39′16″ N	022°04′42″ E	1003 m	*Abies* forest and *Juniperus oxycedrus*; basic soil
CL1159	Greece	Peloponese, Laconia, Monemvasia, Voies Pantanassa	A.R. Burgaz s.n. (MACB 111375)	36°36′40″ N	022°56′58″ E	178 m	Olive grove; acid substrate
CL1165	Greece	Thessaly, Kalabaka, Meterora-Kalithea road	A.R. Burgaz s.n. (MACB 111385)	39°43′35″ N	021°38′55″ E	624 m	Deciduous *Quercus* sp., *Q. coccifera*; acid substrate
CL1183	Greece	Thessaly, Kalabaka, Meterora-Kalithea road	A.R. Burgaz s.n. (MACB 111385)	39°43′35″ N	021°38′55″ E	624 m	Deciduous *Quercus, Q. coccifera*; acid substrate
CL1161	Greece	West Greece, Peloponnese Peninsula, Olympia, Foloi, Kouumanis, Kastania	A.R. Burgaz s.n. (MACB 111378)	37°47′59″ N	021°47′19″ E	646 m	Deciduous *Quercus;* conglomerate and clay substrate
CL1182	Greece	West Greece, Peloponnese Peninsula, Olympia, Foloi, Kouumanis, Kastania	A.R. Burgaz s.n. (MACB 111378)	37°47′59″ N	021°47′19″ E	646 m	Deciduous *Quercus;* conglomerate and clay substrate
CL1156	Italy	Sardinia, Cagliari, Uta, R. Nat. Di Monte Arcosu, near Santa Lucia chapel, strada Guttureddu	A.R. Burgaz s.n. (MACB 110168)	39°12′11″ N	008°55′21″ E	82 m	*Arbutus unedo* and *Pistacia lentiscus*; quartzitic substrate
CL1152	Italy	Sardinia, Sassari, Nuoro, Bolotana, Catena del Maghine	A.R. Burgaz s.n. (MACB 110180)	40°22′02″ N	008°56′18″ E	960 m	*Quercus pubescens* and *Cistus salvifolius*; quartzitic substrate
CL1153	Italy	Sardinia, Sassari, South Sardinia, Arbus, Ingurtosu	A.R. Burgaz s.n. (MACB 110170)	39°30′03″ N	008°31′47″ E	414 m	Garrigue, *Erica arborea*, *Phillyrea angustifolia*, *Ulex* sp., *Cistus monspeliensis*, and *Arbutus unedo*; granitic substrate
CL1155	Italy	Sardinia, Sassari, South Sardinia, Gairo	A.R. Burgaz s.n. (MACB 110169)	39°52′04″ N	009°30′01″ E	982 m	*Quercus rotundifolia*; quartzitic substrate
CL1154	Italy	Sardinia, Sassari, Tempio Pausania Bassacutena	A.R. Burgaz s.n. (MACB 110173)	41°06′54″ N	009°15′45″ E	70 m	Maquis shrubland and *Quercus* sp.; granitic substrate
CL1151	Italy	Sardinia, Sassari, Tempio Pausania Mount Limbara	A.R. Burgaz s.n. (MACB 110178)	40°51′04″ N	009°08′36″ E	1030 m	*Pinus nigra*; granitic substrate
CL1150	Italy	Sardinia, Sassari, Tempio Pausania Mount Limbara, ner Guiardino Botanico di Curadureddu	A.R. Burgaz s.n. (MACB 110174)	40°51′55″ N	009°07′41″ E	569 m	*Pinus pinea*; acid substrate
1IBER *	Portugal	Tras-Os-Montes, Bragança, Macedo de Cavaleiros, Lagoa, Sabor River Valley	R. Pino-Bodas (MACB 93695)	41°25′08″ N	06°45′51″ W	340 m	*Quercus rotundifolia;* quartzitic substrate
CL1044 **	Spain	Toledo, La Nava de Ricomalillo, near Fuentes station	R. Pino-Bodas (MACB 124251)	39°39′51″ N	5°02′04″ W	665 m	*Cistus ladanifer* shrubland; quarzitic substrate
CL1043 **	Spain	Toledo, Sevilleja de la Jara to Anchuras	R. Pino-Bodas (MACB 124250)	39°33′55.0″ N	4°57′39.7″ W	720 m	*Cistus ladanifer* shrubland; quarzitic substrate
2IBER *	Spain	Jaén, Chiclana de Segura, near the Dañador River	A.R. Burgaz (MACB 93537)	38°24′57″ N	02°59′54″ W	743 m	*Quercus rotundifolia;* acid substrate
4IBER *	Spain	Madrid, Manzanares el Real, P. Nat. de La Pedriza, Senda de Quebrantaherraduras	A.R. Burgaz (MACB 100442)				
2SUBT *	Spain	Cordoba, Villaharta, La Lastrilla Spring	A.R. Burgaz (MACB 100445)	38°07′14″ N	04°54′02″ W	515 m	*Quercus rotundifolia;* quartzitic substrate
3SUBT *	Spain	Ciudad Real, Villamanrique, Sierra Morena	A.R. Burgaz (MACB 99488)	38°26′53″ N	03°00′14″ W	741 m	*Quercus rotundifolia;* acid substrate

* Photobiont sequenced using Sanger. ** Specimens used to culture the photobiont.

**Table 2:** Results of the BLAST searches for the nineteen ASVs. The taxonomy of the genus *Trebouxia* follows Muggia et al. [5].

ASV	Taxonomy	N° Reads	Best BLAST Hit	% ID
ASV1	*Myrmecia* sp.	230,708	OL625167	99.64%
ASV2	*Myrmecia* sp.	44,752	OL625167	99.29%
ASV3	*Myrmecia* sp.	12,485	OL625167	99.29%
ASV4	*Myrmecia* sp.	6,947	OL625167	99.29%
ASV5	*Myrmecia* sp.	5,437	OL625167	99.29%
ASV6	*Asterochloris mediterranea*	973	KP257394	100.00%
ASV7	*Trebouxia* sp. (A04)	525	KR912894	100.00%
ASV8	*Vulcanochloris symbiotica*	356	OL625153	100.00%
ASV9	*Trebouxia lynnae* (A39)	210	KY066424	100.00%
ASV10	*Coccomyxa* sp.	188	MN738564	94.85%
ASV11	*Trebouxia* sp. (A04)	177	KR913187	100.00%
ASV12	*Trebouxia lynnae* (A39)	170	MG687518	99.69%
ASV13	*Trebouxia jamesii* (A03)	164	MN397126	100.00%
ASV14	*Trebouxia lynnae* (A39)	141	MG687518	100.00%
ASV15	*Trebouxia maresiae* (A46)	132	MH254833	100.00%
ASV16	*Trebouxia lynnae* (A39)	129	MG687518	99.69%
ASV17	*Hemichloris antarctica*	119	HG972970	88.85%
ASV18	*Trebouxia lynnae* (A39)	105	MG687518	99.69%
ASV19	Uncultured alga	103	ON119386	99.65%

**Table 3:** Results of the indicator species analysis indicate that the ASVs are significantly associated with the countries.

			Country			
ASV	Indicator Statistic	*p*-Value	France	Greece	Italy	Spain
*ASV1*	0.898	0.001				X
*ASV2*	0.543	0.05		X		
*ASV6*	0.671	0.007				X
*ASV7*	0.754	0.002				X
*ASV11*	0.905	0.001				X
*ASV19*	0.933	0.001				X

## Data Availability

All sequences are available in GenBank under OR760202–OR760210 accession numbers and bioproject PRJNA1033642.

## Supplementary Materials

The following supporting information can be downloaded at: https://www.mdpi.com/article/10.3390/jof9121160/s1, Figure S1: Distribution of the main photobiont ASVs in the Mediterranean region. Table S1: Sequences from GenBank included in the phylogenetic analyses with accession numbers, lichen-forming fungi, geographical region, and reference. Table S2: Specimens used in the ultrastructural study with voucher specimens.
