# Supplementary material for: Myrmecia, Not Asterochloris, Is the Main Photobiont of Cladonia subturgida (Cladoniaceae, Lecanoromycetes)
Source: J Fungi (Basel). 2023 Dec 2;9(12):1160. doi: 10.3390/jof9121160 (PMC10744234; doi:10.3390/jof9121160)
Supplement: Supplementary file 1 [file jof-09-01160-s001.zip › jof-2723880-supplementary.pdf]

Table S1. Sequences from GenBank included in the phylogenetic analyses with accession numbers, lichen-forming fungi, geographical region and reference

GenBank	Species name	Mycobiont	Geographic origin	Reference
FJ418565	*Trebouxia lynnae*	*Ramalina farinacea*	Spain	Gasulla et al. 2010
KM020046	*Lobosphaera incisa*	Non lichenized	Austria	Karsten et al. 2005
KR952330	*Vulcanochloris guanchorum*	*Stereocaulon vesuvianum*	Spain (Canary Islands)	Vančurová et al. 2015
KY981643	*Myrmecia israelensis*	*Psora decipiens*	Spain	Moya et al. 2018
KY981645	*Myrmecia israelensis*	*Psora saviczii*	Spain	Moya et al. 2018
KY981647	*Myrmecia israelensis*	*Psora decipiens*	Spain	Moya et al. 2018
KY981651	*Myrmecia israelensis*	*Psora decipiens*	Czech Republic	Moya et al. 2018
KY981653	*Myrmecia israelensis*	*Psora decipiens*	Spain	Moya et al. 2018
KY981655	*Myrmecia israelensis*	*Psora decipiens*	Spain	Moya et al. 2018
KY981657	*Myrmecia israelensis*	*Psora decipiens*	Germany	Moya et al. 2018
KY981659	*Myrmecia israelensis*	*Psora decipiens*	Spain (Canary Islands)	Moya et al. 2018
KY981662	*Myrmecia israelensis*	*Clavascidium* sp.	Spain	Moya et al. 2018
KY981668	*Myrmecia israelensis*	*Clavascidium* sp.	Spain	Moya et al. 2018
KY981669	*Myrmecia israelensis*	Culture (UTEX 1181)	—	Moya et al. 2018
KY981670	*Myrmecia israelensis*	*Placidium* sp.	Czech Republic	Moya et al. 2018
KY981671	*Myrmecia israelensis*	*Psora decipiens*	Croatia	Moya et al. 2018
KY981674	*Myrmecia israelensis*	*Psora decipiens*	Spain	Moya et al. 2018
KY981679	*Myrmecia israelensis*	*Placidium pilosellum*	Slovakia	Moya et al. 2018
KY981681	*Myrmecia israelensis*	*Placidium pilosellum*	Cyprus	Moya et al. 2018
KY981682	*Myrmecia israelensis*	*Psora decipiens*	Slovakia	Moya et al. 2018
KY981683	*Myrmecia israelensis*	*Psora* sp.	Slovakia	Moya et al. 2018
KY981684	*Myrmecia israelensis*	*Placidium* sp.	Slovakia	Moya et al. 2018
KY981685	*Myrmecia israelensis*	*Placidium* sp.	Czech Republic	Moya et al. 2018
KY981686	*Myrmecia israelensis*	*Placidium* sp.	Cyprus	Moya et al. 2018
KY981687	*Myrmecia israelensis*	*Psora decipiens*	Cyprus	Moya et al. 2018
KY981688	*Myrmecia israelensis*	*Placidium* sp.	Cyprus	Moya et al. 2018
KY981693	*Myrmecia israelensis*	*Psora decipiens*	Slovakia	Moya et al. 2018
KY981694	*Myrmecia israelensis*	*Placidium* sp.	Slovakia	Moya et al. 2018
KY981695	*Myrmecia israelensis*	*Psora decipiens*	Turkey	Moya et al. 2018
KY981696	*Myrmecia israelensis*	*Psora decipiens*	Germany	Moya et al. 2018
KY981697	*Myrmecia israelensis*	*Psora decipiens*	Germany	Moya et al. 2018
KY981700	*Myrmecia israelensis*	*Placidium* sp.	Slovakia	Moya et al. 2018

MT581398	*Myrmecia israelensis*	*Acarospora nodulosa*	Spain	Moya et al. 2020
OL625167	*Myrmecia* sp.	*Cladonia* sp.	Spain (Canary Islands)	Vančurová et al. 2021
ON620065	*Myrmecia* sp.	Non lichenized	Argentina	Cometto et al. 2022
ON620066	*Myrmecia* sp.	Non lichenized	Argentina	Cometto et al. 2022
ON620067	*Myrmecia* sp.	Non lichenized	Argentina	Cometto et al. 2022
ON620068	*Myrmecia* sp.	Non lichenized	Argentina	Cometto et al. 2022

Table S2. Specimens used in the untraestructure study.

**Taxa**	**Locality**	**Collector & Collection**
Cladonia humilis s.lat	Spain, Toledo, Belvis de la Jara, open forest of *Quercus rotundifolia*, 39°44'35.5"N 4°58'10.4"W, 13 Oct 2018	R. Pino-Bodas s.n. (MACB 124252)
Cladonia foliacea	Spain, Toledo, Belvis de la Jara, open forest of *Quercus rotundifolia*, 39°44'35.5"N 4°58'10.4"W, 13 Oct 2018	R. Pino-Bodas s.n. (MACB 124248)
Cladonia subturgida	Spain, Toledo, Belvis de la Jara, open forest *of Quercus rotundifolia*, 39°44'35.5"N 4°58'10.4"W, 13 Oct 2018	R. Pino-Bodas s.n. (MACB 124249)

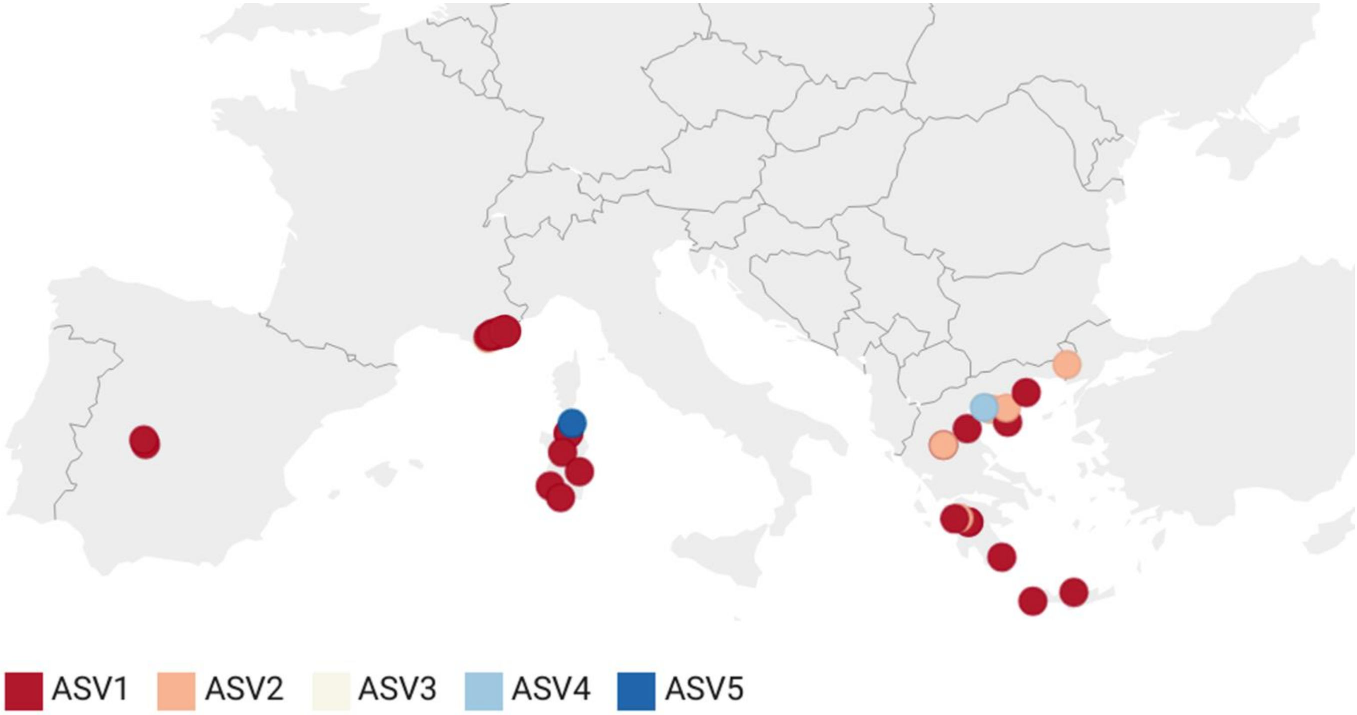

Figure S1. Distribution of the main photobiont ASVs in the Mediterranean region
